# The 5-HTTLPR polymorphism of the serotonin transporter gene and short term behavioral response to methylphenidate in children with ADHD

**DOI:** 10.1186/1471-244X-10-50

**Published:** 2010-06-22

**Authors:** Geeta A Thakur, Natalie Grizenko, Sarojini M Sengupta, Norbert Schmitz, Ridha Joober

**Affiliations:** 1 Integrated Program in Neuroscience, McGill University, (3801 University Street), Montreal, (H3A 2B4), Canada; 2 Department of Psychiatry, McGill University, (1033 Pine Avenue West), Montreal, (H3A 1A1), Canada; 3 Department of Human Genetics, McGill University, (1205 Dr Penfield Avenue), Montreal, (H3A 1B1), Canada; 4 Douglas Mental Health University Institute, (6875 LaSalle Blvd.), Montreal, (H4 H 1R3), Canada

## Abstract

**Background:**

Animal models of ADHD suggest that the paradoxical calming effect of methylphenidate on motor activity could be mediated through its action on serotonin transmission. In this study, we have investigated the relationship between the 5-HTTLPR polymorphism in the serotonin transporter gene (*SLC6A4*) and the response of ADHD relevant behaviors with methylphenidate treatment.

**Methods:**

Patients between ages 6-12 (n = 157) were assessed with regard to their behavioral response to methylphenidate (0.5 mg/kg/day) using a 2-week prospective within-subject, placebo-controlled (crossover) trial. The children were then genotyped with regard to the triallelic 5-HTTLPR polymorphism in the *SLC6A4 *gene. Main outcome measure: Conners' Global Index for parents (CGI-Parents) and teachers (CGI-Teachers) at baseline and at the end of each week of treatment with placebo and methylphenidate. For both outcome measurements, we used a mixed model analysis of variance to determine gene, treatment and gene × treatment interaction effects.

**Results:**

Mixed model analysis of variance revealed a gene × treatment interaction for CGI-Parents but not for CGI-Teachers. Children homozygous for the lower expressing alleles (*s+l_G _= s'*) responded well to placebo and did not derive additional improvement with methylphenidate compared to children carrying a higher expressing allele (*l_A_*). No genotype main effects on either CGI-Parents or CGI-teachers were observed.

**Conclusions:**

A double blind placebo-controlled design was used to assess the behavioral effects of methylphenidate in relation to the triallelic 5-HTTLPR polymorphism of the *SLC6A4 *gene in children with ADHD. This polymorphism appears to modulate the behavioral response to methylphenidate in children with ADHD as assessed in the home environment by parents. Further investigation is needed to assess the clinical implications of this finding.

**Trial Registration:**

ClinicalTrials.gov NCT00483106

## Background

Attention-deficit/hyperactivity disorder (ADHD) is the most common neurodevelopmental disorder in childhood, affecting 8-12% of children worldwide [1]. It is characterized by developmentally inappropriate levels of attention, motor activity and impulsivity. ADHD is characterized by high heritability (0.76) [1], likely conferred by several genes, each making a small contribution to the overall risk for the disorder [1].

It has become common practice in genetic association studies of ADHD to focus on genes implicated in brain monoamine transmission, given that psychostimulant drugs, such as methylphenidate (MPH), are effective in controlling ADHD symptoms. It is widely believed that this effect results primarily, but not exclusively, from modulation of dopamine transmission and downstream monoamine systems. Although this strategy of using a "pharmacological bridge" to select candidate genes is reasonable, it is possible that molecular mechanisms responsible for the pharmacology of psychostimulants and the underlying pathological processes of ADHD do not coincide or coincide only to some extent. Numerous genetic association studies investigating these pharmacological candidates in relation to ADHD have been published. However, results have proven difficult to replicate and the odds ratios associated with each gene were low, ranging from 1.13 to 1.45 (for review see [2]). This may reflect, among other things, the low *a priori *probability that these genes are implicated in ADHD [3]. In contrast, the *a priori *probability that some of these genes are associated with the "drug response phenotype" may be substantially higher given the important biological evidence that psychostimulants interact with different molecular targets in the brain dopamine system and downstream neurotransmission pathways. For example, it has been demonstrated that MPH binds to the dopamine transporter and blocks its activity [4]. Thus, genetic variants that affect the structure and/or the function of the dopamine transporter may retain reasonable *a priori *probability to modulate behavioral response to MPH. In fact, in a recent study [5], we found that the 3'-UTR VNTR polymorphism in the dopamine transporter gene modulates behavioral response to MPH, replicating a previous study by Stein et al. [6]. Additionally, investigating the "drug response" phenotype has the important advantage of being amenable to the placebo-controlled, double blind study design, a robust design for controlling bias in behavioral research studies.

Although the primary mechanism of action of MPH is believed to involve increased synaptic dopamine and norepinephrine levels, it has been suggested that behavioral response to MPH may also be mediated through serotonergic mechanisms acting downstream of the dopamine system. Giros et al. have shown that dopamine transporter gene-knockout mice display a high level of motor activity, a phenotype amplified by exposure to a novel environment [7]. This hyperactivity is reduced with the administration of MPH, even though these animals display high levels of extra-cellular dopamine. Gainetdinov et al. postulated that this paradoxical effect could be mediated by serotonin (5-HT) neurotransmission pathways [8,9]. In accordance with this hypothesis, they have shown that a selective 5-HT transporter inhibitor (fluoxetine) or 5-HT enhancers (5-hydroxytryptophan or L-tryptophan) significantly reduce motor hyperactivity in this animal model but not in wild type animals. In contrast, in the same animal model, it was shown that nisoxitine, a norepinephrine transporter blocker, does not affect locomotor hyperactivity. Thus, it appears that the level of dopaminergic tone may determine the potency of the serotonergic enhancers in decreasing the hyperactivity observed in the dopamine transporter knockout model of ADHD. Notwithstanding some problems in extending these animal findings to children affected with ADHD [10,11], these observations suggest that brain 5-HT pathways may play a role in mediating the pharmacological effect of psychostimulants. Given the critical role of the serotonin transporter (5-HTT) in regulation of 5-HT transmission [12], the 5-HTT gene *(SLC6A4) *may be considered a suitable candidate gene to explain at least part of the variability in behavioral response to MPH.

A polymorphism in the promoter region of the serotonin transporter gene (5-HTTLPR or 5-HTT gene-linked polymorphic region) has been shown to modulate transcriptional activity and availability of 5-HTT both *in vitro *and *in vivo *with the short allele (*s*) having reduced transcriptional activity compared to the long allele (*l*) [12-14]. This polymorphism has been extensively studied in relation to mood/anxiety disorders and impulse dyscontrol related disorders. It is generally believed that the *s *allele (or the *ss *genotype), associated with low levels of transcription of the *SLC6A4*, results in a desensitization of post synaptic 5-HT receptors, which in turn increase the risk for these disorders. More recently, it has been shown that this polymorphism is triallelic, with the *l *allele having two forms, *l_G _*and *l*_A_, resulting from a common G→A polymorphism. The *l_G _*allele creates a functional AP2 transcription-factor binding site. Expression assays showed nearly equivalent expression for the *s *and *l_G _*alleles, accounting for more variation in 5-HTT expression [15]. Previous association studies between ADHD and the 5-HTTLPR polymorphism yielded various results [16-22]. Five case-control association studies reported an association between 5-HTTLPR and ADHD or its symptomatic dimensions [23-27]. These studies suggest that the *l *allele may be associated with externalizing disorders whereas the *s *allele may be related to internalization symptoms. In line with these case-control studies, three family studies reported a trend towards [16,18,21] and one study reported a significant over transmission [17] of the *l *allele of 5-HTTLPR from parents to children with ADHD. The overall risk associated with *SLC6A4 *was estimated to be 1.31 with 95% confidence interval of (1.09-1.59) [2].

Under the assumption that 5-HT transmission may be part of the biological mechanisms mediating behavioral response to MPH, we have examined the association of the polymorphic 5-HTTLPR with behavioral response to MPH by using a double-blind, placebo-controlled crossover study to test this association.

## Methods

### Subjects

One hundred and fifty seven children were recruited from the Disruptive Behavior Disorders Program and the child psychiatry outpatient clinics at the Douglas Institute in Montreal. They were referred to these specialized care facilities by schools, community social workers, family doctors and pediatricians.

To be included in the study, children had to be between 6 and 12 years of age and meet DSM-IV diagnostic criteria for ADHD [28]. Diagnosis was based on observation of the child's behavior and an interview with at least one parent by a child psychiatrist. This clinical examination was supplemented by a structured clinical interview of parents using the DISC-IV (parental report) [29] as well as school reports. In the majority of cases, mothers were the primary informants. Parents completed the Child Behavioral Checklist or CBCL [30], a scale that assesses several behavioral domains of the child. For this scale, parents were asked to give an overall evaluation of the child's behavior without a specific timeframe.

In order to assess behavioral response to MPH, we used the Conners' Global Index for parents (CGI-Parents) and teachers (CGI-Teachers) [31]. Parents and teachers were instructed to assess the child's behavior during the week preceding each assessment. The CGI-Parents and CGI-Teachers are subsets of the original Conners' Rating Scales, widely used for assessing symptoms of ADHD and other psychopathology in children between 3 and 17 years of age for which normative data have been well established [32]. Each Conners' Global Index (CGI) scale consists of 10 items representing the Hyperactivity Index of the original Conners' scale. Each item describes a behavior that is rated on a 4-point Likert scale from 0 (not at all true) to 3 (very true). CGI-Parents and CGI-Teachers are each comprised of two factors: "Emotional lability" and "Restless-impulsive behavior". The raw total and factor scores are transformed into normalized T-scores, with 65 or higher considered to be clinically significant. This rating scale has been recommended for titrating and monitoring treatment with psychostimulant drugs [32]. All these assessments were completed during the week preceding the clinical trial (i.e. at baseline) while the children were not taking any medication.

Children with a history of Tourette's syndrome, bipolar disorder, pervasive developmental disorder, psychosis or any medical condition or impairment that would interfere with the ability of the child to complete the study, were excluded.

### Procedure

Once each child completed the baseline evaluations, a 2-week double-blind, placebo-controlled, within-subject crossover experimental design was used to assess the behavioral response to MPH as compared to placebo. Since the primary purpose of the study was to investigate the effect of the *SLC6A4 *genotype on the variability of behavioral response to MPH, we used a moderate dose (0.5 mg/kg/day) of MPH that has been shown to produce a robust behavioral response in most children [33]. This dose also follows the recommendations of initiating treatment with MPH at a low to moderate dose and titrating to higher doses if the child does not respond adequately [34]. In addition, although several studies reported a linear dose-response curve of behaviors to MPH [35,36], other studies suggested a curvilinear dose-response curve, with higher doses adding only a marginal effect compared to medium doses [37]. Thus, we reasoned that using a 0.5 mg/kg/day is an adequate dose to study behavioral response to MPH within its linear dose-response curve segment. After one week of baseline assessments, which also served as a wash-out period for children previously treated with MPH, subjects received one week of treatment with placebo followed by one week of treatment with 0.5 mg/kg/day of MPH given in a divided dose (morning and noon). The order of administration (placebo and MPH) was blind, counterbalanced and determined by random assignment.

Placebo and MPH were prepared individually in opaque gelatin capsules in weekly blister packs by a pharmacist not otherwise involved in the study to maintain the blind allocation of treatment. At the end of each week of treatment, the blister packs were collected and medication compliance checked. At this time, a research assistant contacted the child's parents and teacher and asked them to fill the CGI-Parents and CGI-Teachers respectively, taking into consideration the behavior of the child during the entire previous week of treatment (including weekends for parents).

The research protocol was approved by the Research Ethics Board of the Douglas Institute. Parents provided written informed consent. The study was explained to the children and they gave their verbal assent to participate.

### Molecular genetics

The 5-HTTLPR polymorphism of the *SLC6A4 *gene was genotyped using PCR amplification of DNA and resolution of different alleles using agarose gel electrophoresis according to previously published methods [38]. The *l_G _*and *l_A _*alleles were subsequently studied by enzymatic digestion of 7 μl of the above mentioned PCR product using 5 U of MspI and incubation at 37°C for a minimum of 3 hours. The *l_G _*and *l_A _*alleles were then resolved on a 2% agarose gel. Because the *l_G _*and *s *alleles were shown to be functionally equivalent, we derived three new genotype groups: *s's' *(*l_G _l_G_*, *s l_G_*, and *ss*), *s'l' *(*l_G _l_A _*and *s l_A_*) and *l'l' *(*l_A _l_A_*).

### Statistical analyses

Our primary outcome variables were CGI-Parents and CGI-Teachers. The effects of genotype (*s's'*, *s'l' *and *l'l'*), gender, treatment (placebo and MPH), treatment order and genotype by treatment interaction were tested using a mixed model analysis of variance with an unstructured covariance matrix (SAS MIXED procedure, SAS version 6.12, SAS Institute Inc, Cary, NC) [39]. Treatment, order of treatment, genotype, and treatment by genotype interaction were fixed effects; individuals were random effects. The mixed model analysis of variance has many advantages over repeated measures analysis of variance [40]. Main effects and any interactions were regarded as statistically significant when p < 0.05. Baseline value of the outcome scores (CGI-Parents or CGI-Teachers ratings) were respectively included as a covariate [41]. For significant genotype by treatment interaction, simple contrasts were carried out to explore how genotype and treatment interact.

Demographic and clinical characteristics of the three 5-HTTLPR genotype groups were compared using ANOVA or χ^2 ^tests as appropriate.

## Results

The distribution of the 5-HTTLPR genotypes [*ss *(23%), *sl *(45%), *ll *(32%)] did not depart from Hardy Weinberg proportions (χ^2 ^= 0.63, df = 2, p = 0.73). Similarly, *s's' *(27%), *s'l' *(51%) and *l'l' *(22%) genotype distribution did not depart from Hardy Weinberg proportions (χ^2 ^= 0.62, df = 2, p = 0.73).

### 5-HTTLPR and clinical characteristics

Clinical characteristics of the three groups of children are presented in Table 1. The distribution of the common comorbid disorders of ADHD (oppositional defiant disorder, conduct disorder, anxiety disorders and mood disorders) were similar among the three groups of children, although anxiety disorders tended to be more frequent in children with the *s's' *and, to a lesser extent, *s'l' *genotypes compared to children with the *l'l' *genotype. However, females were more represented in the group of children with the *s's' *genotype (p = 0.008). Thus, we controlled for gender in subsequent analyses.

**Table 1:** Baseline characteristics of children with ADHD separated according to genotype in the triallelic 5-HTTLPR polymorphism of the *SLC6A4 *gene.

	*s's' *genotype (n = 42)	*s'l' *genotype (n = 81)	*l'l' *genotype (n = 34)	Statistic and p-value
Age	8.9 (1.80)	9.2 (1.90)	8.8 (1.70)	F_2,154 _= 0.7, p = 0.52
				
Males/Females (% males)	30/12 (78%)	67/14 (88.5%)	34/0 (87.5%)	χ^2 ^= 11.2, df = 2, p = 0.003
				
Household income (% < $20,000 per year)	41.5%	31.6%	44.8%	χ^2^= 1.9, df = 2, p = 0.38
Ethnic origin (Caucasian/non-Caucasian)	35/7	72/9	30/4	χ^2 ^= 0.8, df = 2, p = 0.92
WISQ-III full scale IQ	96.9 (15.4)	99.3 (15.2)	97.3 (14.5)	F_2,144 _= 0.4, p = 0.69
				
CBCL				
Total score	69.4 (9.4)	69.5 (10.3)	70.4 (6.9)	F_2,149 _= 0.1, p = 0.88
Internalizing problems	65.2 (11.8)	64.5 (12.3)	62.4 (8.6)	F_2,149 _= 0.6, p = 0.54
Withdrawn	64.1 (11.7)	64.2 (10.7)	61.4 (7.6)	F_2,149 _= 0.9, p = 0.41
Somatic complaints	61.3 (8.5)	59.3 (8.5)	56.9 (7.0)	F_2,149 _= 2.4, p = 0.10
Anxiety/depression	65.1 (10.6)	66.3 (11.9)	63.0 (8.8)	F_2,149 _= 1.1, p = 0.34
Social problems	65.8 (10.6)	67.6 (10.6)	68.0 (10.6)	F_2,149 _= 0.5, p = 0.60
Thought problems	63.0 (11.1)	63.5 (10.8)	62.5 (9.5)	F_2,149 _= 0.1, p = 0.88
Attention problems	70.3 (11.3)	70.5 (9.9)	68.3 (9.6)	F_2,149 _= 0.6, p = 0.56
Externalizing problems	69.3 (9.5)	69.2 (11.4)	72.8 (7.6)	F_2,149 _= 1.6, p = 0.21
Aggressive behavior	71.4 (11.9)	72.9 (13.6)	75.1 (9.7)	F_2,149 _= 0.8, p = 0.46
Diagnosis C/I/H	21/16/5	42/30/9	25/5/4	χ^2 ^= 6.6, df = 4, p = 0.15
				
Comorbidity (%) with				
CD	28.5%	28.5%	30.5%	χ^2 ^= 0.03, df = 2, p = 1.0
ODD	38.0%	37.5%	42.5%	χ^2 ^= 0.2, df = 2, p = 0.88
AD	44.5%	28.0%	19.0%	χ^2 ^= 5.4, df = 2, p = 0.07
MD	16.7%	13.3%	3.8%	χ^2 ^= 2.4, df = 2, p = 0.30
				
Previously medicated (%)	51.3%	46.7%	48.4%	χ^2 ^= 0.22, df = 2, p = 0.90

Values are mean (SD) unless otherwise indicated. Demographic and clinical characteristics were compared between these groups using the appropriate statistic depending on the nature of the data. WISC = Wechsler Intelligence Scale for Children, 3rd edition; CBCL = Child Behavioral Checklist; C/I/H = Combined/Inattentive/Hyperactive; ODD = Oppositional Defiant Disorder, CD = Conduct Disorder, AD = Anxiety Disorder, MD = Major Mood Disorder. Number of observations varied some times with regard to variables (i.e. CBCL, WISC-III). Variation n number of observation is reflected in the degrees of freedom.

### Behavioral response to clinical intervention

A significant behavioral response during the week of treatment with placebo, as assessed by comparing CGI scores during the placebo week over baseline measures, was observed for CGI-Parents (F_1, 156 _= 166, p < 0.001) and CGI-Teachers (F_1, 156 _= 16.4, p < 0.001). This indicates that the clinical intervention, irrespective of whether an active drug or a placebo is delivered, resulted in a robust behavioral response compared to the baseline levels as assessed by both parents and teachers.

### Effect of 5-HTTLPR on behavioral response to MPH

**Figure 1:**
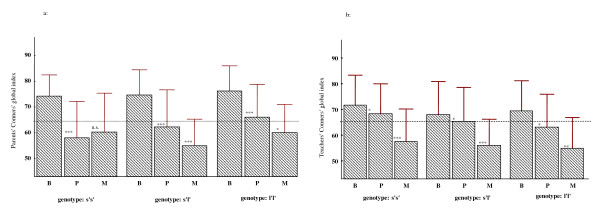
**CGI-P and CGI-T scores separated according to child's triallelic 5-HTTLPR genotype at three time points (B, P, and M)**. Conners' global index scores (± SD) for parents (1a) and teachers (1b) in children with ADHD separated according to their genotype in the 5-HTTLPR of the serotonin transporter gene (*SLC6A4*) during baseline evaluation (B), treatment with placebo (P) and treatment with methylphenidate (M). Dashed line represents the threshold for clinical significance on the Conners' scales (≥ 65). Asterisks indicate the levels of significance of the differences in CGI scores between different assessments (B *vs*. P and P *vs*. M). ***: p < 0.001, **: p < 0.01, *: p < 0.05, ns = non significant.

Mixed model analysis of variance with CGI-P scores as the dependent variable, genotype as the independent variable, and baseline scores and gender as the covariates showed a significant genotype by treatment 2-way interaction [F_2, 152 _= 5.43, p = 0.005]. As depicted in Figure 1a, behavioral response to MPH as compared to placebo was significant for both the *l'l' *[F_1, 33 _= 5.3, p = 0.028] and *s'l' *[F_1, 80 _= 23.9, p < 0.000] genotype groups. The *s's' *group showed no significant response to treatment with MPH [F_1, 41 _= 0.66, p = 0.42]. We also calculated the change scores between the week of treatment with placebo and the week of treatment with MPH. These scores were analyzed using univariate analysis of variance, which revealed a significant genotype effect [F_2, 154 _= 6.0, p = 0.003]. *Post hoc *comparisons of the mean difference scores using the Tukey's Honestly significant differences (HSD) test revealed significant differences between the *s's' *and *s'l' *(p = 0.002) and between the *s's' *and *l'l' *(p = 0.04) genotype groups. There were no differences between *s'l' *and *l'l' *genotype groups (p = 0.88). When we performed similar analyses using a recessive model where the independent between-subject variable was restricted to two genotype categories (*s's' vs. s'l'+l'l'*), a highly significant genotype effect was also observed (F_1, 155 _= 11.9, p = 0.0007). These results remained significant when we controlled for anxiety disorders (p values < 0.05).

Similar analyses comparing baseline scores to placebo also revealed a genotype effect [F_2, 154 _= 4.15, p = 0.02]. The *s's' *genotype group displayed a significant improvement with placebo compared to the *s'l' *genotype group (p = 0.02). No differences between *s's' *and *l'l' *or *s'l' *and *l'l' *genotype groups were observed.

For the CGI-T scores, the mixed model analysis of variance revealed a highly significant treatment effect [F_1, 152 _*= *31.0, p < 0.0001], no main effect of genotype [F_2, 152 _*= *1.29, p *= *0.28], nor a genotype by treatment interaction [F_2, 152 _*= *0.18, p *= *0.83] (Figure 1b).

### Effect of 5-HTTLPR on side effects induced by MPH

Side effects were collected in 105 patients both during the week of treatment with MPH and the week of treatment with placebo, using the Barkley Stimulant Drug Side Effects Rating Scale [42], which delineates 17 side effects commonly reported during treatment with stimulant medications. Parents assessed the presence and severity of these side effects, rating each item on a scale of 0 (absent) to 9 (severe). For each of these side effects, we calculated the change score between the week of treatment with placebo and the treatment with MPH. None of the side effects was modulated by 5-HTTLPR genotype at a Bonferroni corrected level of significance (0.003). All p-values were higher than 0.15, except for the item "talks less" (p = 0.03). In a post-hoc analysis (Tukey's HSD), patients with the *s's' *genotype tended to talk less than patients with the *s'l' *(p = 0.06) and patients with the *l'l' *(p = 0.06) genotypes after treatment with MPH. There was no correlation between CGI-Parents and "talks less" change scores (r = -0.12, p = 0.19), indicating that this item did not affect the parental rating of response to MPH.

### Interaction with the Dopamine transporter VNTR polymorphism

In order to investigate the interaction between the 3'-UTR VNTR in the *SLC6A3 *gene and the 5-HTTLPR in the *SLC6A4 *gene, we conducted an ANOVA where genotypes in each of these two genes were the independent factor and the change score in CGI-Parents was the dependent outcome variable. This analysis resulted in a significant effect of the 5-HTTLPR genotype (F_2, 112 _= 5.13, p = 0.007), a marginal effect of the DAT genotype (F_2, 112 _= 2.35, p = 0.09) but no gene by gene interaction.

## Discussion

Using MPH to probe behaviors in children with ADHD may be a good approach to investigate relations between genes and behaviors in this disorder. This approach has the advantage of being supported by a wealth of information on the molecular and neurochemical mechanisms of MPH. The use of the double blind placebo-controlled design is likely to refine the quality of the pharmaco-behavioral genetic approach by controlling for bias in the evaluation of behaviors. It is notable in this regard that the only three studies using a double blind placebo-controlled design to investigate behavioral response to amphetamine in healthy volunteers [43] and MPH in children with ADHD [5,6] found convergent results indicating that subjects homozygous for the 9-repeat allele in the 3'UTR VNTR polymorphism of the dopamine transporter gene are less sensitive to the effects of psychostimulants.

Based on the hypothesis of Gainetdinov and Caron [8,9], that the paradoxical calming effects of MPH may be due to enhanced 5-HT transmission downstream of the dopamine system, it is possible that patients who are homozygous for the *s' *allele, presumed to be impaired with regard to 5-HT transmission, would present poor response to MPH. In contrast, patients homozygous for the *l' *allele, presumed to have more efficient 5-HT transmission, would show very good response to MPH.

The main finding of this study is consistent with this hypothesis to a certain extent. Indeed, we identified an association between the triallelic 5-HTTLPR polymorphism in the 5-HTT gene and behavioral response to MPH as assessed by the CGI- Parents. Children with the *s's' *genotype responded significantly better to placebo and did not derive a further improvement with MPH. On the contrary, children with the *l'l' *genotype responded minimally to placebo and derived significant improvement with MPH. Children with the *s'l' *genotype had an intermediate profile. The statistical differences are also reflected in clinical differences. Indeed, children with the *l'l' *genotype retained clinically significant impairment after treatment with placebo (CGI-Parents>65) and improved to the non-clinical range with MPH. In contrast, children with the *s's' *genotype showed significant clinical improvement with placebo and appeared to deteriorate when MPH was added to their treatment.

Studies investigating the pharmacogenetics of ADHD have reported both positive and negative results. Some recent findings have detected no significant associations between polymorphisms of the DAT1, DRD4 and 5-HTT genes and response to MPH [44,45]. However, it has been difficult to compare results from these studies given significant variability in key methodological issues.

This current study design however is potentially more robust given the placebo arm, which acts as an internal control, quantitative measures of MPH response by two raters, namely parents and teachers, as well as consideration of comorbid disorders.

On the other hand, we did not identify a genotype or genotype-by-treatment interaction with therapeutic response to MPH as assessed by teachers in the school environment. Although this apparently contradicts findings from the parents' evaluation, this result could be interpreted by the fact that environmental factors and observer effects, as well as the interaction between the observer and the child have an important impact on the child's behavior and its assessment [46]. In fact, genetic epidemiological studies suggest that the genetic factors implicated in ADHD symptoms as evaluated by parents and teachers are at least partially different [47,48] (see review by Thapar et al.[49]). Also, it has been demonstrated that parent and teacher ratings of improvement with MPH correlate only modestly [50] and differ in their magnitude [33]. Faraone et al. reported that when poor or no response is reported by one reporter (parents or teachers), it is unlikely to be confirmed by the other [50]. Thus, it is possible that the 5-HTTLPR may be specifically modulating behaviors and therapeutic response of these behaviors to MPH as evaluated by parents in the home environment.

Also, it should be noted that the effect of the 5-HTTLPR genotype remained significant after controlling for the effect of the 3'UTR VNTR polymorphism of the dopamine transporter gene, which was previously reported to be associated with response to MPH [5,6]. However, these results need to be replicated in a larger sample given that some genotype combinations were represented by few subjects.

Some limitations should be kept in mind while interpreting the results from this study. First, this study used a single dose of MPH (0.5 mg/kg/day), which is in the low to medium range commonly used in clinical practice. As our primary purpose was to use MPH as a pharmacological probe to challenge behaviors relevant for ADHD, we used a dose that is both in the low/medium therapeutic range and recommended as a starting dose. Even though this dose elicited clinically and statistically significant improvements, an experimental design with several doses (including lower doses for some genotypes) and longer periods of treatment with MPH treatment will be important to further explore the clinical implications of the present findings. Second, our sample comprised relatively few females. Separate analyses on males and females revealed essentially the same results in males but no significant findings in females. A larger sample needs to be studied in order to validate or refute these results in girls.

Furthermore, although the majority of subjects were Caucasians, it is possible that differences in other loci which are differentially distributed between the three genotype groups could confound this finding. However, there were no significant differences in the distribution of Caucasians/non-Caucasians between the three genotype groups.

## Conclusion

In conclusion, this pharmacogenetic study investigating the role of the 5-HTT gene in behavioral response to MPH in children with ADHD is the largest study using the double-blind, placebo-controlled, crossover design for the evaluation of behavioral response to psychostimulants. In addition, the assessment tools used in this study were developed for children, have been standardized with regard to the general population and were shown to be adequate for the evaluation of therapeutic response to drugs in children with ADHD. The results of this study strongly suggest that children with the *s's', s'l' *and *l'l' *genotypes may be characterized with differential profiles of response to placebo and MPH in the context of a short term clinical trial. If these profiles are confirmed in a larger group of patients, this may help in the design of better interventions that are tailored for the specific needs of each of these groups of patients.

## Competing interests

Dr. Ridha Joober receives a consultation honorarium from Janssen-Ortho and Pfizer Canada.

## Authors' contributions

GT analyzed results and prepared the manuscript. NG helped design the study, assessed the patients, evaluated therapeutic response and provided conceptual input on the manuscript content. SS carried out molecular genetic studies and provided conceptual input on the manuscript content. NS conducted the statistical analyses. RJ designed the study, assessed the patients, and supervised the conception and writing of the manuscript. All authors have read and approved the final manuscript.

## Pre-publication history

The pre-publication history for this paper can be accessed here:

http://www.biomedcentral.com/1471-244X/10/50/prepub
